# Comparative efficacy and safety of Chinese medicine injections as an adjunctive therapy for cervical cancer in Chinese patients: a network meta-analysis

**DOI:** 10.1080/13880209.2024.2312217

**Published:** 2024-02-09

**Authors:** Fei Ma, Qun Wang, Di Zhang, Zihong Wang, Hui Xie, Xianghong Liu, Hongxing Zhang, Haiyan Song, Shiguang Sun

**Affiliations:** a Office of Party Committee (Director), Affiliated Hospital, Shandong Provincial Hospital of Chinese Medicine, Shandong University of Traditional Chinese Medicine, Jinan, China; b College of Traditional Chinese Medicine, Shandong University of Traditional Chinese Medicine, Jinan, China; c Department of Vertigo, Jinan Shizhong People’s Hospital, Jinan, China; d College of Pharmacy, Shandong University of Traditional Chinese Medicine, Jinan, China; e Department of Pharmacy, Qilu Hospital, Shandong University, Jinan, China; f Department of Pharmacy, Jinan Hospital of Chinese Medicine, Shandong University of Traditional Chinese Medicine, Jinan, China; g Department of Pharmacy, Second Affiliated Hospital, Shandong Provincial Hospital of Integrated Medicine, Shandong University of Traditional Chinese Medicine, Jinan, China

**Keywords:** Chinese medicine injections (CMIs), Radiotherapy (RT), concurrent chemoradiotherapy (CCRT), efficacy, safety, network meta-analysis, randomized controlled trials (RCTs)

## Abstract

**Context:**

Chinese medicine injections (CMIs) are widely used as adjuvant therapy for cervical cancer in China. However, the effectiveness of different types of CMIs remains uncertain.

**Objective:**

To assess the effectiveness and safety of CMIs when used in conjunction with radiotherapy (RT) or concurrent chemoradiotherapy (CCRT), particularly in combination with cisplatin (DDP), docetaxel plus cisplatin (DP), and paclitaxel plus cisplatin (TP).

**Materials and methods:**

Randomized controlled trials (RCTs) were searched in databases including CNKI, WanFang, VIP, SinoMed, PubMed, Cochrane Library, Embase, and Web of Science from inception to September 2023. We calculated the risk ratio with a 95% confidence interval and the surface under the cumulative ranking area curve (SUCRA) for the clinical efficacy rate (CER), the efficacy rate by Karnofsky Performance Status (KPS), and the rates of leukopenia reduction (LRR) and gastrointestinal reactions (GRR).

**Results:**

Forty-seven RCTs were included, including nine CMI types: *Aidi*, *Fufangkushen*, *Huangqi*, *Kangai* (KA), *Kanglaite* (KLT), *Renshenduotang*, *Shenqifuzheng* (SQFZ), *Shenmai* (SM), and *Yadanzi*. KLT and KA were likely optimal choices with radiotherapy for CER and KPS, respectively. KA and KLT were optimal choices with RT + DDP for CER and GRR, respectively. KLT was the likely optimal choice with RT + DP for CER and KA for both KPS and GRR. SM and SQFZ were the likely optimal choices with RT + TP for CER and LRR, respectively.

**Conclusions:**

The optimal recommendation depends on whether CMIs are used with radiotherapy or concurrent chemoradiotherapy. More high-quality RCTs are needed to confirm further and update the existing evidence.

## Introduction

Globally, cervical cancer is the fourth most common female malignant tumor in both incidence and mortality, ranking only after breast cancer, colorectal cancer, and lung cancer, which is centrally caused by persistent human papillomavirus (HPV) infections, with HPV strains 16 and 18 causing the vast majority of cases (Sung et al. 2021). Due to the existence of a transparent socioeconomic gradient in cervical cancer, the incidence is three times higher in countries with a low Human Development Index (HDI) compared to countries with a very high HDI. At the same time, the mortality is six times higher in low HDI countries than in countries with very high HDI (Singh et al. 2023). As the most populous country, China is suffering from a high burden of cervical cancer; the trend in incidence of cervical cancer increased from 1990 to 2019, particularly in younger age, while the trend in mortality declined in older age (Shen et al. 2022).

Chronic infection by high-risk HPV oncogenic subtypes causes almost all cases of cervical cancer. Therefore, effective primary prevention of cervical cancer depends on HPV detection and vaccination (Johnson et al. 2019). Meanwhile, secondary prevention and treatment depend on the extent of the disease at diagnosis and locally available resources and may involve radical hysterectomy, chemoradiation, or a combination of both (Cohen et al. 2019). Radiotherapy (RT), especially brachytherapy, is the primary treatment in the management of cervical cancer for early-stage tumors with local risk factors. At the same time, concurrent chemoradiotherapy (CCRT) is the standard treatment for advanced local tumors (Chargari et al. 2022). Various combinations of cisplatin, paclitaxel, bevacizumab, carboplatin, topotecan, and gemcitabine are recommended as first-line therapies (Koh et al. 2015). Meanwhile, a recent meta-analysis reported that cisplatin-based CCRT, especially paclitaxel plus cisplatin (TP), could be the best choice for cervical cancer in efficacy and safety (Fu et al. 2017; Li 2022).

Traditional Chinese medicine (TCM), recognized as a complementary and alternative medical system, is among the oldest healthcare systems globally. Chinese medicine injections (CMIs), notably, representative anticancer CMIs within Chinese patent medicines, have found extensive use in the clinical management of cervical cancer in China over many years. However, the effectiveness of specific types of CMIs, particularly in combination with RT or CCRT for cervical cancer, remains uncertain. The current study conducted a network meta-analysis to comprehensively evaluate the efficacy and safety of CMIs as adjuvant treatments in conjunction with RT or cisplatin-based CCRT for managing cervical cancer. This evaluation was compared to conventional treatment alone to provide evidence and guidance for clinical medication strategies.

## Materials and methods

We followed the Preferred Reporting Items for Systematic Reviews and Meta-Analyses (PRISMA) guidelines (Page et al. 2021), and this study was registered with PROSPERO (CRD42022361708).

### Search strategy

We searched the literature in four Chinese databases (CNKI, WanFang, VIP, and SinoMed), and four English databases (PubMed, Cochrane Library, Embase, and Web of Science), from inception to September 2023. The search strategy employed encompassed the terms "cervical cancer", "radiotherapy", and "chemoradiotherapy" along with their respective abbreviations and all synonymous variations tailored for each specific database.

### Eligibility criteria

#### Inclusion criteria

1. Type of population: patients diagnosed with cervical cancer.
2. Type of intervention: combined with CMIs.
3. Type of comparison: RT or cisplatin-based CCRT, such as cisplatin (DDP), docetaxel plus cisplatin (DP), and TP.
4. Type of outcomes: efficacy evaluation indicators were the clinical efficacy rate (CER) and efficacy rate according to the Karnofsky performance status (KPS). Safety evaluation indicators were the leukopenia reduction rate (LRR) and gastrointestinal reaction rate (GRR). CER was evaluated using the Response Evaluation Criteria in Solid Tumors (RECIST) (Therasse et al. 2000). KPS was examined according to the Karnofsky performance scale score, in which an increase of ≥ 10 after treatment is considered an improvement, a decrease of ≥10 is regarded as a decrease, and stability is considered between the two (Yates et al. 1980). LRR and GRR were evaluated for incidence and severity using the Common Terminology Criteria for Adverse Events (CTCAE) (Trotti et al. 2003). These outcomes were calculated using the following formula: CER = number of complete and partial response patients/total number of patients × 100%; KPS = number of patients who responded to improvement by the KPS score/total number of patients × 100%; LRR = (number of patients with leukopenia reduction adverse events/total number of patients) × 100%; GRR = (number of patients with gastrointestinal adverse events/total number of patients) × 100%.
5. Type of study design: randomized controlled trials (RCTs).

#### Exclusion criteria

1. Duplicates.
2. Non-clinical trial study.
3. Non-randomized controlled study.
4. The control group or treatment group combined traditional Chinese medicine therapies such as footbath, acupuncture catgut embedding, acupoint application, and others.

### Literature screening and data extraction

After removing duplicates of retrieved studies, the two screening phases were conducted by two independent reviewers (F Ma and Q Wang): (1) titles and abstracts were screened according to the eligible criteria; (2) full texts of initial studies were further screened for the final inclusion. The following information were extracted: study characteristics (authors, publication year, country), patient characteristics (age, tumor stage), interventions (drug, treatment duration), comparisons and outcomes. Any disagreements were solved by the third author (H Xie).

### Assessment of methodological quality

The methodological quality assessment was conducted by two independent reviewers (D Zhang or ZH Wang) using the Cochrane Bias Risk Assessment Tool, which was composed of randomization, protocol concealment, allocation concealment, measurement blinding, completeness of outcome data, selective outcome reporting, and other biases (Higgins et al. 2011). To evaluate the risk of bias of studies, each item was rated as low risk, high risk, or unclear risk. Any disagreements were solved by the third author (H Xie).

### Statistical analysis

The frequentist random-effect model with STATA 15.0 software was adopted to perform the network meta-analysis (Chaimani et al. 2013; Chaimani and Salanti 2015). The risk ratio (RR) with a 95% confidence interval (95% CI) was calculated for binary data. The surface under the cumulative ranking area curve (SUCRA) was calculated to rank multiple interventions. SUCRA values of 100% and 0% were assigned as the best and worst treatments for efficacy and safety, respectively. A sensitivity analysis was conducted to assess the reliability. Finally, a comparison-adjusted funnel plot was created to evaluate the publication bias and the effect of small studies (Wang and Sun 2020).

## Results

### Selection of the included studies

**Figure 1.**
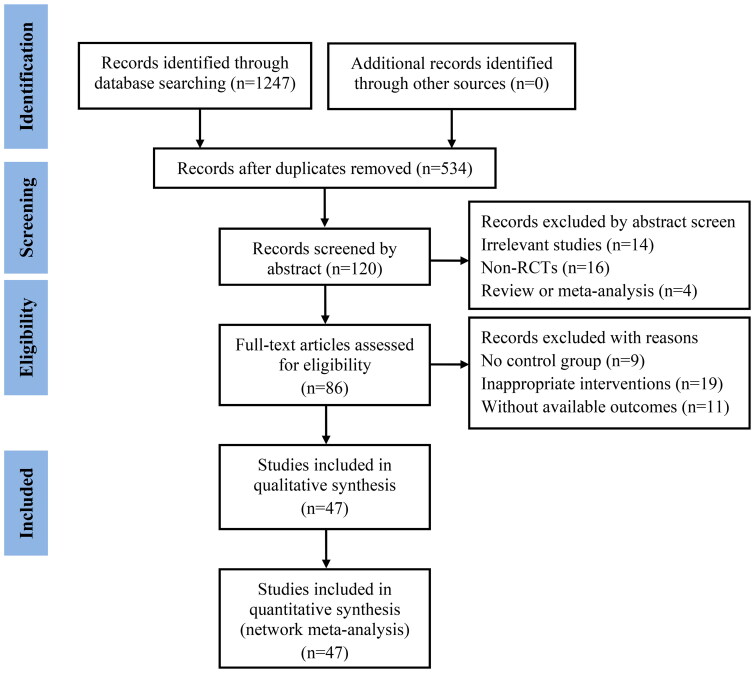
The fYlow diagram of study selection.

According to the search strategy, 47 eligible studies (Cao and Wen 2005; Liu 2009; Hu and Lei 2011; Lu et al. 2011; Wang et al. 2011; Yin et al. 2011; Zhou et al. 2011; Chen and Liu 2012; Chen and Zhang 2012; Wen et al. 2012; Chen 2013; Wu et al. 2013; Chen et al. 2014; Deng and Cai 2014; Deng and Chen 2014; Huang et al. 2014; Li et al. 2014; Zhong et al. 2014; Li et al. 2015; Liu et al. 2015; Deng et al. 2015a, 2015b; Jiang et al. 2016; Li et al. 2016; Mu 2016; Qin et al. 2016; Xiong and Zhou 2016; Gao et al. 2017; Li 2017; Zhang 2017; Li et al. 2018; Li and Wu 2018; Ma et al. 2018; Shao 2018; Wang et al. 2018; Yan et al. 2018; Cao 2019; Tian et al. 2019; Zhang 2019; Dong et al. 2020; Li et al. 2020; Liu et al. 2020; Li and Xi 2020; Song et al. 2020; Wang 2021; Ge et al. 2022; Peng and Li 2022) were included, involving 4138 cases, 4 types of comparisons (RT, RT + DDP, RT + DP, RT + TP) and 9 types of CMIs, such as *Aidi* (AD), *Fufangkushen* (FFKS), *Huangqi* (HQ), *Kangai* (KA), *Kanglaite* (KLT), *Renshenduotang* (RSDT), *Shenqifuzheng* (SQFZ), *Shenmai* (SM), and *Yadanzi* (YDZ). The study selection is shown in Figure 1.

### Characteristics of the included studies

All 47 studies were single-center trials conducted in China and published from 2005 to 2022. Among these CMIs, AD was mentioned in 3 studies, FFKS in 15 studies, HQ in 2 studies, KA in 8 studies, KLT in 5 studies, RSDT in 1 study, SQFZ in 2 studies, SM in 2 studies, and YDZ in 9 studies. Among these comparisons and interventions, RT was mentioned in 17 studies while RT + AD in 1 study, RT + FFKS in 5 studies, RT + KA in 2 studies, RT + KLT in 1 study, RT + YDZ in 8 studies; RT + DDP in 8 studies while RT + DDP + AD in 2 studies, RT + DDP + KA in 2 studies, RT + DDP + KLT in 2 studies, RT + DDP + FFKS in 2 studies; RT + DP in 10 studies while RT + DP + KA in 3 studies, RT + DP + KLT in 2 studies, RT + DP + FFKS in 4 studies, and RT + DP + RSDT in 1 study. RT + TP was mentioned in 11 studies while RT + TP + FFKS in 4 studies, RT + TP + HQ in 2 studies, RT + TP + KA in 1 study, RT + TP + SM in 2 studies, RT + TP + SQFZ in 1 study, and RT + TP + YDZ in 1 study. The treatment duration for all studies ranged from 2 to 28 weeks (Table 1).

**Table 1.** The main characteristics of the included randomized controlled trials.

Study	Country	Sample size		Age		Stage (FIGO)	Interventions		Duration (days)	Outcomes	
		Control	Experimental	Control	Experimental		Control	Experimental		Efficacy	Safety
Cao and Wen (2005)	China	53	57	31–72	35–74	II-IV	RT	RT + KA	40	CER, KPS	LRR
Liu (2009)	China	42	42	28–65	30–70	Ia-IIa	RT	RT + KA	30	CER, KPS	–
Hu and Lei (2011)	China	34	73	45–75	45–75	IIb-IV	RT	RT + AD	42	CER, KPS	LRR
Lu et al. (2011)	China	33	33	58.1 ± 9.5	60.4 ± 11.6	II-III	RT	RT + FFKS	63	CER, KPS	–
Wang et al. (2011)	China	23	23	43–75	35–72	–	RT	RT + FFKS	28	CER, KPS	–
Yin et al. (2011)	China	43	46	30-–75	32–78	IIb-IIIb	RT	RT + FFKS	28	CER, KPS	LRR, GRR
Wu et al. (2013)	China	32	28	33–72	29–71	Ib-IIa	RT	RT + YDZ	28	CER	–
Deng and Cai (2014)	China	34	34	33–66	33–66	IIb-IIIb	RT	RT + YDZ	21	CER	LRR
Deng and Chen (2014)	China	34	34	33-66	33-66	IIb-IIIb	RT	RT + YDZ	21	CER	–
Chen et al. (2014)	China	34	34	33–66	33–66	IIb-IIIb	RT	RT + YDZ	21	CER	–
Deng et al. (2015a)	China	34	34	33–66	33–66	IIb-IIIb	RT	RT + YDZ	21	–	LRR
Deng et al. (2015b)	China	35	35	33–65	32–64	IIb-IIIb	RT	RT + FFKS	21	CER	–
Liu et al. (2015)	China	40	40	60–71	60–73	I-IIb	RT	RT + YDZ	84	KPS	–
Yan et al. (2018)	China	30	30	41–68	33–65	IIb-IIIb	RT	RT + FFKS	42	CER, KPS	LRR
Li et al. (2018)	China	40	40	–	–	IIa-IV	RT	RT + YDZ	28	KPS	–
Tian et al. (2019)	China	75	75	22–50	22–50	IIb-IIIb	RT	RT + KLT	35	CER, KPS	LRR, GRR
Li and Xi (2020)	China	39	39	60–70	60–70	I-IIb	RT	RT + YDZ	21	CER, KPS	–
Zhou et al. (2011)	China	28	28	35–66	35–66	IIb-IV	RT + DDP	RT + DDP + AD	28	CER, KPS	LRR, GRR
Li et al. (2014)	China	40	40	25–70	25–70	IIb-IVa	RT + DDP	RT + DDP + KA	30	CER, KPS	GRR
Zhong et al. (2014)	China	34	34	31–59	33–62	III-IV	RT + DDP	RT + DDP + KA	42	CER, KPS	LRR, GRR
Xiong and Zhou (2016)	China	64	64	31–71	27–72	IIb-IV	RT + DDP	RT + DDP + FFKS	42	CER	–
Qin et al. (2016)	China	30	30	40–76	42–77	IIb-IIIb	RT + DDP	RT + DDP + FFKS	28	CER, KPS	LRR
Li and Wu (2018)	China	30	30	30–60	28–59	IIa-IIIb	RT + DDP	RT + DDP + KLT	25	CER	–
Wang (2021)	China	49	49	71–84	70–85	III-IV	RT + DDP	RT + DDP + KLT	84	CER, KPS	LRR, GRR
Ge et al. (2022)	China	49	49	53–72	48–76	II-IV	RT + DDP	RT + DDP + AD	56-82	CER	GRR
Chen and Liu (2012)	China	36	42	31–61	35–59	IIIa-IV	RT + DP	RT + DP + KA	28	CER, KPS	LRR, GRR
Chen and Zhang (2012)	China	34	42	33–61	31–60	III-IV	RT + DP	RT + DP + RSDT	84	CER, KPS	LRR, GRR
Chen (2013)	China	30	30	36–61	32–64	III-IV	RT + DP	RT + DP + FFKS	84	CER, KPS	LRR, GRR
Li et al. (2015)	China	50	50	20–60	20–60	IIb-IVb	RT + DP	RT + DP + KLT	28	CER	–
Mu (2016)	China	100	100	57.1 ± 2.3	55.6 ± 2.5	IIIa-IV	RT + DP	RT + DP + FFKS	54	CER, KPS	LRR, GRR
Jiang et al. (2016)	China	40	40	42–67	40–65	IIIa-IV	RT + DP	RT + DP + FFKS	84	CER, KPS	LRR, GRR
Ma et al. (2018)	China	33	32	40–65	40–65	Ib-IIa	RT + DP	RT + DP + SQFZ	63	–	LRR, GRR
Wang et al. (2018)	China	51	51	30–65	32–63	Ia-IIb	RT + DP	RT + DP + KA	42	CER	GRR
Zhang (2019)	China	50	50	21–60	20–60	–	RT + DP	RT + DP + KLT	196	CER	–
Liu et al. (2020)	China	45	45	40–65	41–63	IIb-IIIb	RT + DP	RT + DP + FFKS	84	CER, KPS	GRR
Song et al. (2020)	China	50	50	35–70	35–70	IIb-IIIb	RT + DP	RT + DP + KA	56	CER	GRR
Wen et al. (2012)	China	32	33	>30	>30	IIb-IVa	RT + TP	RT + TP + SM	15	CER, KPS	LRR, GRR
Huang et al. (2014)	China	33	35	30–64	30–64	IIb-IV	RT + TP	RT + TP + SQFZ	21	CER, KPS	LRR, GRR
Li et al. (2016)	China	55	55	30–60	28–59	Ib-IIb	RT + TP	RT + TP + HQ	10	CER	–
Gao et al. (2017)	China	100	100	40–69	41–70	–	RT + TP	RT + TP + SM	21-35	CER	–
Li (2017)	China	32	32	31–64	31–65	III-IV	RT + TP	RT + TP + FFKS	28	CER	LRR, GRR
Zhang (2017)	China	60	60	31–75	30–74	IIb-IV	RT + TP	RT + TP + KA	56	CER	LRR, GRR
Shao (2018)	China	20	20	42–67	40–68	IIIa-IV	RT + TP	RT + TP + FFKS	63	CER	LRR, GRR
Cao (2019)	China	60	60	38–68	41–72	–	RT + TP	RT + TP + FFKS	30	CER	LRR, GRR
Dong et al. (2020)	China	37	37	35–66	35–65	IIIa-IV	RT + TP	RT + TP + FFKS	63	CER	GRR
Li et al. (2020)	China	65	65	42–52	44–54	Ib2-IIb	RT + TP	RT + TP + HQ	42	CER	LRR, GRR
Peng and Li (2022)	China	48	48	51.2 ± 3.4	49.9 ± 3.5	IIb-IIIb	RT + TP	RT + TP + YDZ	21	CER	LRR, GRR

FIGO: Federation of International of Gynecologists and Obstetricians; RT: radiotherapy; DDP, cisplatin chemotherapy; DP: docetaxel plus cisplatin chemotherapy; TP: paclitaxel plus cisplatin chemotherapy; AD: *Aidi*; HQ, *Huangqi*; FFKS: *Fufangkushen*; KA: *Kangai*; KLT: *Kanglaite*; RSDT: *Renshenduotang*; SQFZ: *Shenqifuzheng*; SM: *Shenmai*; YDZ: *Yadanzi*; CER: clinical efficacy rate; KPS: Karnofsky performance scale rate; LRR: leukocyte reduction rate; GRR: gastrointestinal reaction rate; -, not reported.

### Quality assessment of the included studies

**Figure 2.**
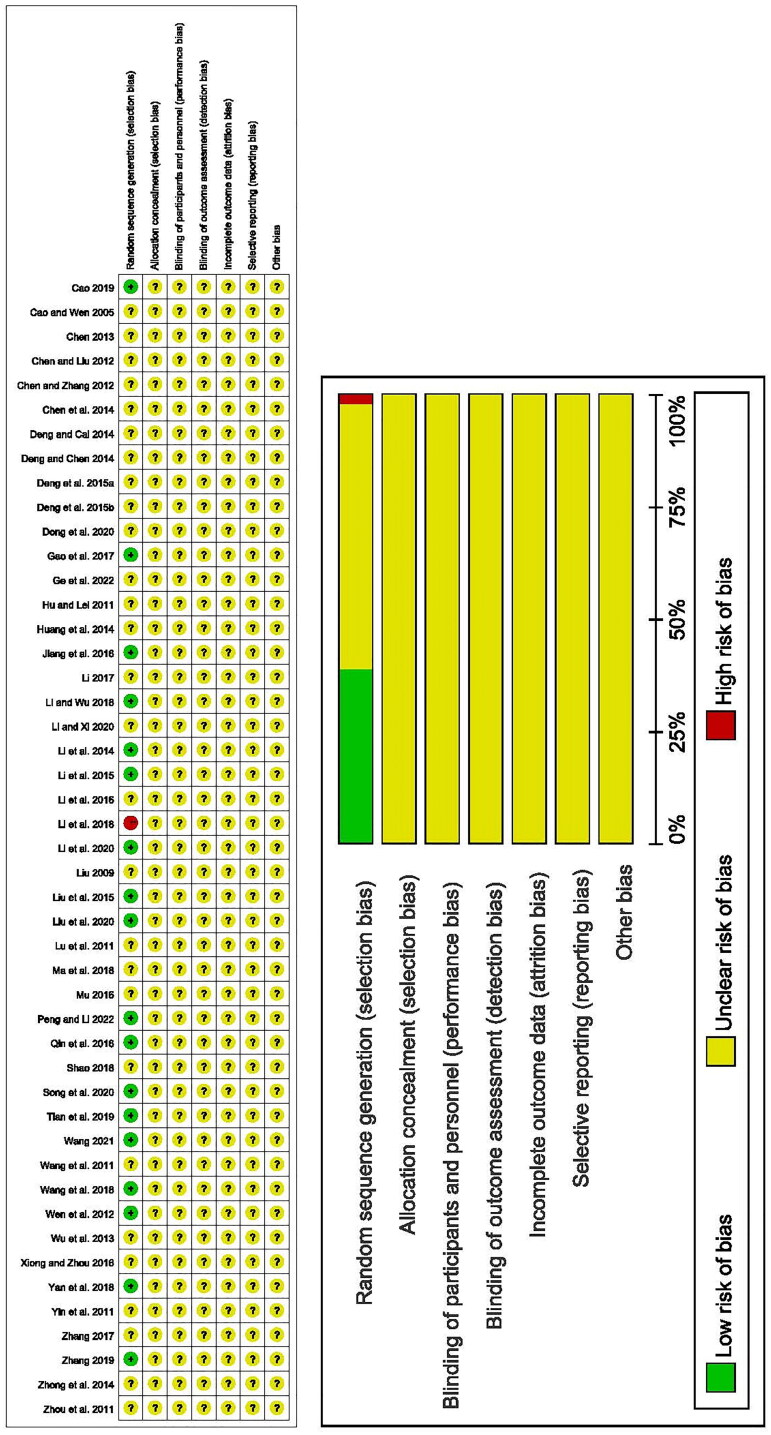
Risk of bias graph of included randomized controlled trials.

Among the 47 studies included, only 18 studies provided details about the randomization method. Of these, 17 studies were evaluated as low-risk, and 1 as high-risk. The remaining studies did not provide specific information on the details of the randomization. None of the studies mentioned protocol concealment, allocation concealment, measurement blinding, completeness of outcome data, selective outcome reporting, or other biases. As a result, all of these aspects were rated as unclear risk (Figure 2)

### Network meta-analysis

#### Clinical efficacy rate (CER)

**Figure 3.**
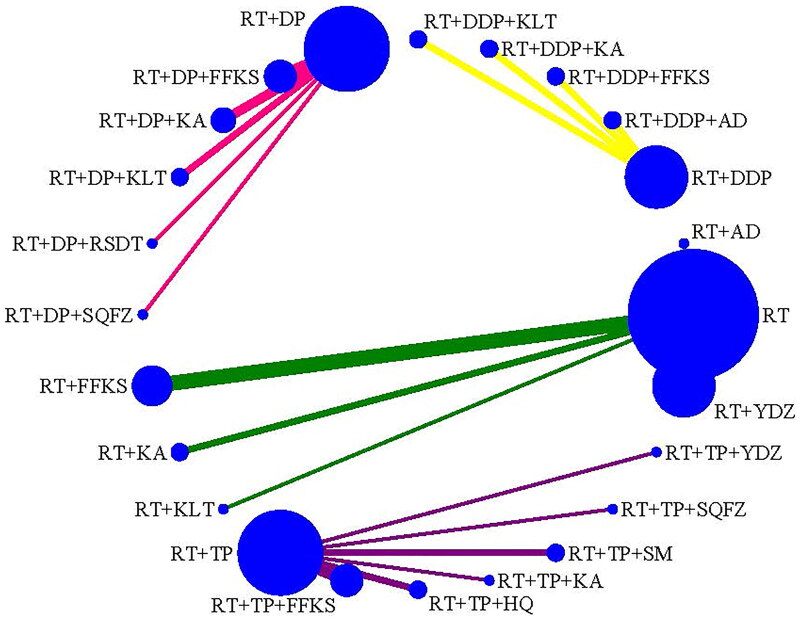
Network plot for the efficacy (CER) of CMIs combined with RT or CCRT for cervical cancer.

CER was reported in 43 studies (Cao and Wen 2005); Liu 2009; Hu and Lei 2011; Lu et al. 2011; Wang et al. 2011; Yin et al. 2011; Zhou et al. 2011; Chen and Liu 2012; Chen and Zhang 2012; Wen et al. 2012; Chen 2013; Wu et al. 2013; Chen et al. 2014; Deng and Cai 2014; Deng and Chen 2014; Huang et al. 2014; Li et al. 2014; Zhong et al. 2014; Li et al. 2015; Deng et al. 2015b; Jiang et al. 2016; Li et al. 2016; Mu 2016; Qin et al. 2016; Xiong and Zhou 2016; Gao et al. 2017; Li 2017; Zhang 2017; Li et al. 2018; Li and Wu 2018; Shao 2018; Wang et al. 2018; Yan et al. 2018; Cao 2019; Tian et al. 2019; Zhang 2019; Dong et al. 2020; Li et al. 2020; Liu et al. 2020; Song et al. 2020; Wang 2021; Ge et al. 2022; Peng and Li 2022), with 3847 cases, including 4 types of comparisons (RT, RT + DDP, RT + DP, RT + TP) and 5 types of CMIs combined with RT, 4 types of CMIs combined with RT + DDP, 4 types of CMIs combined with RT + DP, 6 types of CMIs combined with RT + TP (Figure 3).

The pooled results indicated that RT + AD, RT + FFKS, RT + KA, and RT + KLT were associated with a significant increase in CER compared to RT alone. Furthermore, RT + DDP + KA and RT + DDP + KLT significantly increased the CER compared to RT + DDP. In the case of RT + DP, RT + DP + FFKS, RT + DP + KA, and RT + DP + KLT, the CER significantly increased compared to RT + DP. For RT + TP, both RT + TP + FFKS and RT + TP + SM significantly increased CER compared to RT + TP. However, RT + TP + HQ was associated with a significant decrease in CER compared to RT + TP + FFKS, and RT + TP + SM was found to substantially increase CER compared to RT + TP + HQ (Table 2).

**Table 2.** Network meta-analysis for the efficacy of CMIs combined with RT or CCRT for cervical cancer.

	KPS						
CER	RT	2.51 (0.42, 15.07)	3.35 (1.00, 11.26)	**9.49 (1.76, 51.17)**	–	1.74 (0.54, 5.63)	
	**2.12 (1.23, 3.65)**	RT + AD	1.33 (0.15, 11.58)	3.77 (0.32, 44.12)	–	0.69 (0.08, 5.89)	
	**1.91 (1.11, 3.29)**	0.90 (0.42, 1.95)	RT + FFKS	2.83 (0.36, 22.55)	–	0.52 (0.10, 2.81)	
	**2.40 (1.20, 4.82)**	1.14 (0.47, 2.75)	1.26 (0.52, 3.04)	RT + KA	–	0.18 (0.02, 1.41)	
	**4.75 (1.67, 13.52)**	2.25 (0.69, 7.31)	2.48 (0.76, 8.07)	1.98 (0.56, 6.94)	RT + KLT	–	
	1.70 (0.88, 3.28)	0.80 (0.34, 1.89)	0.89 (0.38, 2.09)	0.71 (0.27, 1.84)	0.36 (0.10, 1.23)	RT + YDZ	
	RT + DDP	3.00 (0.28, 32.67)	7.00 (0.49, 99.70)	2.82 (0.51, 15.62)	3.45 (0.28, 42.52)		
	1.72 (0.68, 4.33)	RT + DDP + AD	2.33 (0.07, 83.01)	0.94 (0.05, 17.75)	1.15 (0.04, 36.79)		
	1.47 (0.62, 3.52)	0.86 (0.24, 3.06)	RT + DDP + FFKS	0.40 (0.02, 9.50)	0.49 (0.01, 19.07)		
	**3.06 (1.38, 6.75)**	1.78 (0.53, 6.03)	2.07 (0.46, 6.73)	RT + DDP + KA	1.22 (0.06, 25.53)		
	**2.49 (1.21, 5.12)**	1.45 (0.45, 4.69)	1.69 (0.54, 5.23)	0.81 (0.28, 2.38)	RT + DDP + KLT		
	RT + DP	**3.63 (2.10, 6.27)**	**20.50 (2.51, 167.62)**	–	1.29 (0.37, 4.42)		
	**2.12 (1.44, 3.13)**	RT + DP + FFKS	5.65 (0.64, 49.57)	–	0.35 (0.09, 1.37)		
	**2.54 (1.44, 4.48)**	1.20 (0.60, 2.38)	RT + DP + KA	–	**0.06 (0.01, 0.72)**-		
	**5.76 (2.23, 14.84)**	2.71 (0.97, 7.56)	2.27 (0.75, 6.85)	RT + DP + KLT			
	2.15 (0.86, 5.42)	1.02 (0.37, 2.77)	0.85 (0.29, 2.51)	0.37 (0.10, 1.40)	RT + DP + RSDT		
	RT + TP	–	–	–	3.38 (0.13, 89.57)	2.96 (0.48, 18.21)	–
	**3.98 (1.66, 9.56)**	RT + TP + FFKS	–	–	–	–	–
	0.95 (0.33, 2.72)	**0.24 (0.06, 0.94)**	RT + TP + HQ	–	–	–	–
	4.64 (0.96, 22.54)	1.17 (0.19, 7.11)	4.90 (0.73, 32.74)	RT + TP + KA	–	–	–
	**6.96 (2.18, 22.25)**	1.75 (0.41, 7.51)	**7.35 (1.53, 35.28)**	1.50 (0.21, 10.66)	RT + TP + SM	0.87 (0.13, 5.86)	–
	2.96 (0.58, 15.09)	0.74 (0.12, 4.73)	3.13 (0.45, 21.75)	0.64 (0.07, 6.17)	0.43 (0.06, 3.14)	RT + TP + SQFZ	–
	2.74 (0.55, 13.77)	0.69 (0.11, 4.33)	2.89 (0.42, 19.89)	0.59 (0.06, 5.65)	0.39 (0.05, 2.88)	0.93 (0.09, 9.17)	RT + TP + YDZ

RT: radiotherapy; CCRT: concurrent chemoradiotherapy; DDP: cisplatin chemotherapy; DP :docetaxel plus cisplatin chemotherapy; TP: paclitaxel plus cisplatin chemotherapy; AD: *Aidi*; HQ: *Huangqi*; FFKS: *Fufangkushen*; KA: *Kangai*; KLT, *Kanglaite*; RSDT: *Renshenduotang*; SQFZ: *Shenqifuzheng*; SM: *Shenmai*; YDZ: *Yadanzi*; CER: clinical efficacy rate; KPS: Karnofsky performance scale rate; LRR: leukocyte reduction rate; GRR: gastrointestinal reaction rate; -, not reported; The table data on the lower left of the gray bottom tables represents CER, and the upper right KPS; bold font data represents a significant difference.

Based on the SUCRA values for CER, KLT was identified as the most probable to be the best combination with RT. For RT + DDP, KA emerged as the most likely to be the best combination. In the case of RT + DP, KLT was deemed the most probable to be the best combination. Last, for RT + TP, SM was identified as the most likely to be the best combination. The ranking of CMI interventions was as follows: RT + KLT (92.1%) > RT + KA (63.7%) > RT + AD (54.5%) > RT + FFKS (47.9%) > RT + YDZ (39.4%) > RT (2.4%); RT + DDP + KA (83.9%) > RT + DDP + KLT (72.2%) > RT + DDP + AD (47.5%) > RT + DDP + FFKS (38.2%) > RT + DDP (8.2%); RT + DP + KLT (95.7%) > RT + DP + KA (59.7%) > RT + DP + RSDT (47.9%) > RT + DP + FFKS (45.4%) > RT + DP (1.3%); and RT + TP + SM (80.7%) > RT + TP + KA (67.9%) > RT + TP + FFKS (64.1%) > RT + TP + SQFZ (53.7%) > RT + TP + YDZ (50.7%) > RT + TP + HQ (16.9%) > RT + TP (15.9%) (Table 4).

**Table 4.** Rank of SUCRA for the efficacy and safety of CMIs combined with RT or CCRT for cervical cancer.

Therapy	efficacy		safety	
	CER	KPS	LRR	GRR
RT	2.4	17.0	15.2	51.7
RT + AD	54.5	50.1	28.2	–
RT + FFKS	47.9	59.2	71.3	26.2
RT + KA	63.7	86.7	38.8	–
RT + KLT	92.1	–	51.7	72.1
RT + YDZ	39.4	37.1	94.8	–
RT + DDP	8.2	21.3	39.9	0.4
RT + DDP + AD	47.5	51.8	49.5	62.1
RT + DDP + FFKS	38.2	71.2	53.9	–
RT + DDP + KA	83.9	50.8	48.7	46.6
RT + DDP + KLT	72.2	54.8	58.1	90.9
RT + DP	1.3	11.5	29.2	4.3
RT + DP + FFKS	45.4	66.6	34.9	45.9
RT + DP + KA	59.7	97.6	53.6	88.0
RT + DP + KLT	95.7	–	78.5	46.9
RT + DP + RSDT	47.9	24.4	53.7	64.9
RT + TP	15.9	23.8	24.5	33.9
RT + TP + FFKS	64.1	–	12.9	49.6
RT + TP + HQ	16.9	–	68.8	41.9
RT + TP + KA	67.9	–	19.9	58.6
RT + TP + SM	80.7	63.3	78.0	55.8
RT + TP + SQFZ	53.7	62.9	82.1	57.7
RT + TP + YDZ	50.7	–	63.9	52.5

RT: radiotherapy; CCRT: concurrent chemoradiotherapy; DDP: cisplatin chemotherapy; DP: docetaxel plus cisplatin chemotherapy; TP: paclitaxel plus cisplatin chemotherapy; AD: *Aidi*; HQ: *Huangqi*; FFKS: *Fufangkushen*; KA: *Kangai*; KLT: *Kanglaite*; RSDT: *Renshenduotang*; SQFZ: *Shenqifuzheng*; SM: *Shenmai*; YDZ: *Yadanzi*; LRR: leukocyte reduction rate; GRR, gastrointestinal reaction rate; -, not reported.

#### Efficacy rate by Karnofsky Performance Status (KPS)

**Figure 4.**
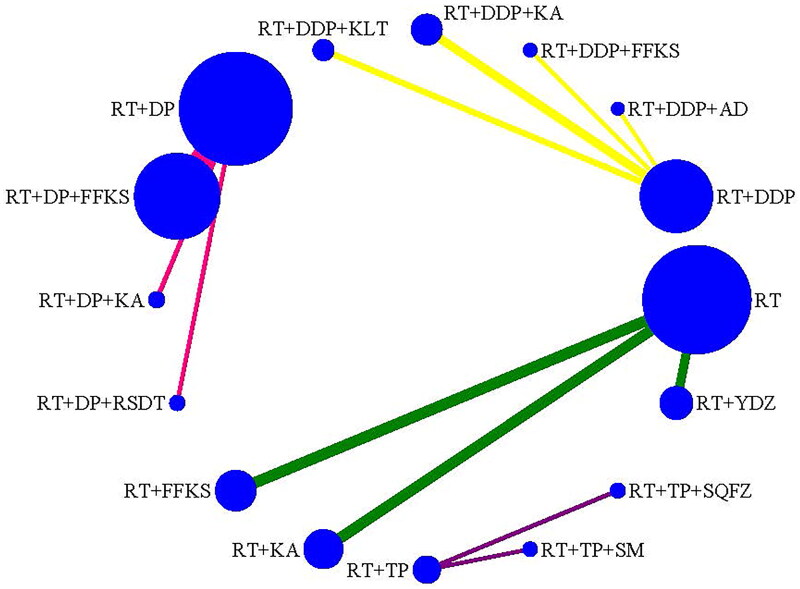
Network plot for the efficacy (KPS) of CMIs combined with RT or CCRT for cervical cancer.

KPS was reported in 24 studies (Cao and Wen 2005; Liu 2009; Hu and Lei 2011; Lu et al. 2011; Wang et al. 2011; Yin et al. 2011; Zhou et al. 2011; Chen and Liu 2012; Chen and Zhang 2012; Wen et al. 2012; Chen 2013; Huang et al. 2014; Li et al. 2014; Zhong et al. 2014; Liu et al. 2015; Jiang et al. 2016; Mu 2016; Qin et al. 2016; Li et al. 2018; Yan et al. 2018; Tian et al. 2019; Liu et al. 2020; Li and Xi 2020; Wang 2021) with 1646 cases, including 4 types of comparisons (RT, RT + DDP, RT + DP, RT + TP) and 5 types of CMIs combination with RT, 4 types of CMIs combination with RT + DDP, 4 types of CMIs combination with RT + DP, and 6 types of CMIs combination with RT + TP (Figure 4).

The pooled results indicated that RT + KA significantly improved KPS compared to RT alone. Additionally, RT + DP + FFKS and RT + DP + KA were associated with a significant increase in KPS compared to RT + DP. In contrast, RT + DP + RSDT decreased the KPS significantly compared to RT + DP + KA (Table 2).

According to the SUCRA values for KPS, KA was the most likely to be the best combination with RT. FFKS was the most likely to be the best combination with RT + DDP. KA was the most likely to be the best combination with RT + DP, and SM was the most likely to be the best combination with RT + TP. The ranking of CMI interventions was as follows: RT + KA (86.7%) > RT + FFKS (59.2%) > RT + AD (50.1%) > RT + YDZ (37.1%) > RT (17%); RT + DDP + FFKS (71.2%) > RT + DDP + KLT (54.8%) > RT + DDP + AD (51.8%) > RT + DDP + KA (50.8%) > RT + DDP (21.3%); RT + DP + KA (97.6%) > RT + DP + FFKS (66.6%) > RT + DP + RSDT (24.4%) > RT + DP (11.5%); RT + TP + SM (63.3%) > RT + TP + SQFZ (62.9%) > RT + TP (23.8%) (Table 4).

#### Rate of leukopenia reduction (LRR)

**Figure 5.**
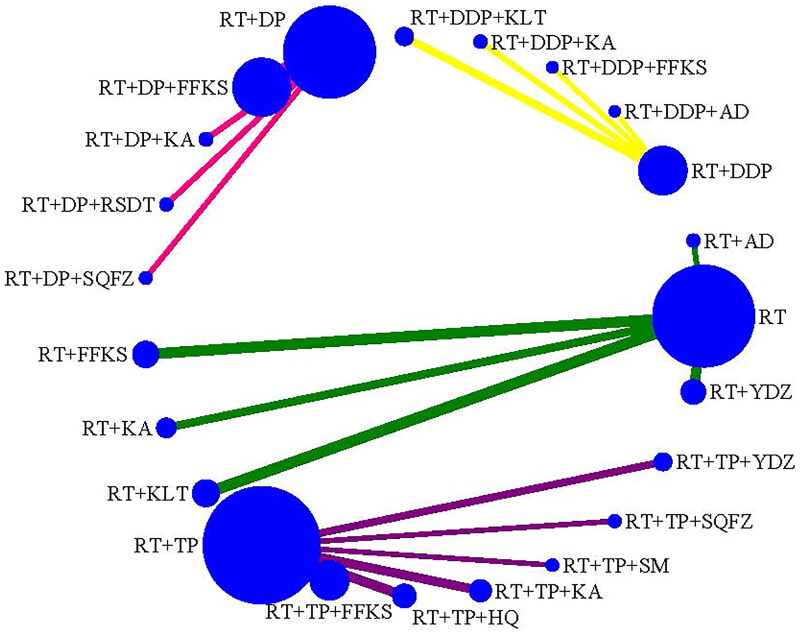
Network plot for the safety (LRR) of CMIs combined with RT or CCRT for cervical cancer.

LRR was reported in 25 studies (Cao and Wen 2005 Hu and Lei 2011; Yin et al. 2011; Zhou et al. 2011; Chen and Liu 2012; Chen and Zhang 2012; Wen et al. 2012; Chen 2013; Chen et al. 2014; Deng and Cai 2014; Huang et al. 2014; Zhong et al. 2014; Deng et al. 2015a; Jiang et al. 2016; Mu 2016; Qin et al. 2016; Li 2017; Zhang 2017; Ma et al. 2018; Shao 2018; Yan et al. 2018; Cao 2019; Tian et al. 2019; Li et al. 2020; Wang 2021; Peng and Li 2022) with 2063 cases, including 4 types of comparisons (RT, RT + DDP, RT + DP, RT + TP) and 5 types of CMIs combination with RT, 4 types of CMIs combination with RT + DDP, 4 types of CMIs combination with RT + DP, and 6 types of CMIs combination with RT + TP (Figure 5).

The pooled results indicate that combinations such as RT + TP + HQ, RT + TP + SM, and RT + TP + SQFZ significantly reduced LRR compared to RT + TP. Furthermore, RT + TP + SM and RT + TP + SQFZ substantially decreased LRR compared to RT + TP + FFKS. RT + TP + SQFZ significantly reduced LRR compared to RT + TP + KA (Table 3).

**Table 3.** Network meta-analysis for the safety of CMIs combined with RT or CCRT for cervical cancer.

	GRR						
LRR	RT	–	2.93 (0.29, 29.53)	–	0.33 (0.01, 17.66)	–	
	0.63 (0.15, 2.62)	RT + AD	–	–	–	–	
	0.10 (0.00, 0.48)	0.17 (0.02, 1.30)	RT + FFKS	–	0.11 (0.00, 4.27)	–	
	0.42 (0.07, 2.62)	0.66 (0.06, 6.86)	3.98 (0.36, 44.13)	RT + KA	–	–	
	0.14 (0.04, 1.56)	0.38 (0.04, 4.02)	2.32 (0.21, 25.83)	0.58 (0.04, 8.08)	RT + KLT	–	
	0.01 (0.00, 0.21)	0.02 (0.00, 0.48)	0.14 (0.01, 3.06)	0.03 (0.00, 0.91)	0.06 (0.00, 1.58)	RT + YDZ	
	RT + DDP	**0.36 (0.19, 0.70)**	–	**0.46 (0.24, 0.89)**	**0.20 (0.07, 0.58)**		
	0.31 (0.00, 254)	RT + DDP + AD	–	–	–		
	0.15 (0.00, 2.52e + 06)	0.49 (0.00, 24961)	RT + DDP + FFKS	1.27 (0.50, 3.21)	0.54 (0.15, 1.93)		
	0.34 (0.00, 903)	1.07, (0.00, 4055)	2.19 (0.00, 1.21e + 07)	RT + DDP + KA	0.43 (0.12, 1.53)		
	0.12 (0.00, 61.88)	0.37 (0.00, 269)	0.77 (0.00, 1.73e + 06)	0.35 (0.00, 2666)	RT + DDP + KLT		
	RT + DP	**0.48 (0.31, 0.75)**	**0.24 (0.14, 0.42)**	0.49 (0.13, 1.78)	0.35 (0.12, 1.03)		
	0.84 (0.19, 3.76)	RT + DP + FFKS	0.51 (0.25, 1.03)	1.02 (0.26, 4.00)	0.74 (0.23, 2.35)		
	0.38 (0.03, 5.08)	0.46 (0.02, 8.98)	RT + DP + KA	2.01 (0.50, 8.11)	1.45 (0.44, 4.80)		
	0.08 (0.00, 3.55)	0.10 (0.00, 5.59)	0.21 (0.00, 20.50)	RT + DP + KLT	0.72 (0.14, 3.88)		
	0.38 (0.03, 5.13)	0.45 (0.02, 9.04)	0.98 (0.03, 38.59)	4.65 (0.05, 457.47)	RT + DP + RSDT		
	RT + TP	0.54 (0.13, 2.26)	0.78 (0.06, 10.07)	0.36 (0.03, 4.71)	0.39 (0.03, 5.59)	0.38 (0.03, 5.41)	0.46 (0.03, 6.23)
	1.40 (0.53, 3.74)	RT + TP + FFKS	1.43 (0.08, 26.78)	0.66 (0.03, 12.50)	0.72 (0.04, 14.68)	0.70 (0.03, 14.21)	0.85 (0.04, 16.49)
	**0.38 (0.16, 0.90)**	0.27 (0.07, 1.00)	RT + TP + HQ	0.46 (0.01, 17.42)	0.50 (0.01, 20.19)	0.49 (0.01, 19.56)	0.60 (0.02, 22.91)
	1.14 (0.48, 2.71)	0.82 (0.22, 3.01)	3.00 (0.89, 10.16)	RT + TP + KA	1.09 (0.03, 44.12)	1.06 (0.03, 42.75)	1.29 (0.03, 50.07)
	**0.29 (0.09, 0.91)**	**0.21 (0.05, 0.94)**	0.77 (0.18, 3.20)	0.26 (0.06, 1.07)	RT + TP + SM	0.97 (0.02, 41.71)	1.19 (0.03, 48.88)
	**0.26 (0.08, 0.80)**	**0.19 (0.04, 0.82)**	0.68 (0.17, 2.81)	**0.23 (0.06, 0.93)**	0.89 (0.18, 4.41)	RT + TP + SQFZ	1.22 (0.03, 49.90)

RT: radiotherapy; CCRT: concurrent chemoradiotherapy; DDP: cisplatin chemotherapy; DP: docetaxel plus cisplatin chemotherapy; TP: paclitaxel plus cisplatin chemotherapy; AD: *Aidi*; HQ: *Huangqi*; FFKS, *Fufangkushen*; KA: *Kangai*; KLT: *Kanglaite*; RSDT: *Renshenduotang*; SQFZ: *Shenqifuzheng*; SM: *Shenmai*; YDZ: *Yadanzi*; GRR: gastrointestinal reaction rate; -, not reported; The table data on the lower left of the gray bottom tables represents LRR, and the upper right GRR; bold font data represents a significant difference.

According to the SUCRA values for LRR, YDZ was the most likely to be the best combination with RT. KLT was most likely to be the best combination with RT + DDP. KLT was the most likely to be the best combination with RT + DP, and SQFZ was the most likely to be the best combination with RT + TP. The ranking of CMIs interventions was as follows: RT + YDZ (94.8%) > RT + FFKS (71.3%) > RT + KLT (51.7%) > RT + KA (38.8%) > RT + AD (28.2%) > RT (15.2%); RT + DDP + KLT (58.1%) > RT + DDP + FFKS (53.9%) > RT + DDP + AD (49.5%) > RT + DDP + KA (48.7%) > RT + DDP (39.9%); RT + DP + KLT (78.5%) > RT + DP + RSDT (53.7%) > RT + DP + KA (53.6%) > RT + DP + FFKS (34.9%) > RT + DP (29.2%); RT + TP + SQFZ (82.1%) > RT + TP + SM (78.0%) > RT + TP + HQ (68.8%) > RT + TP + YDZ (63.9%) > RT + TP (24.5%) > RT + TP + KA (19.9%) > RT + TP + FFKS (12.9%) (Table 4).

#### Rate of gastrointestinal reactions (GRR)

**Figure 6.**
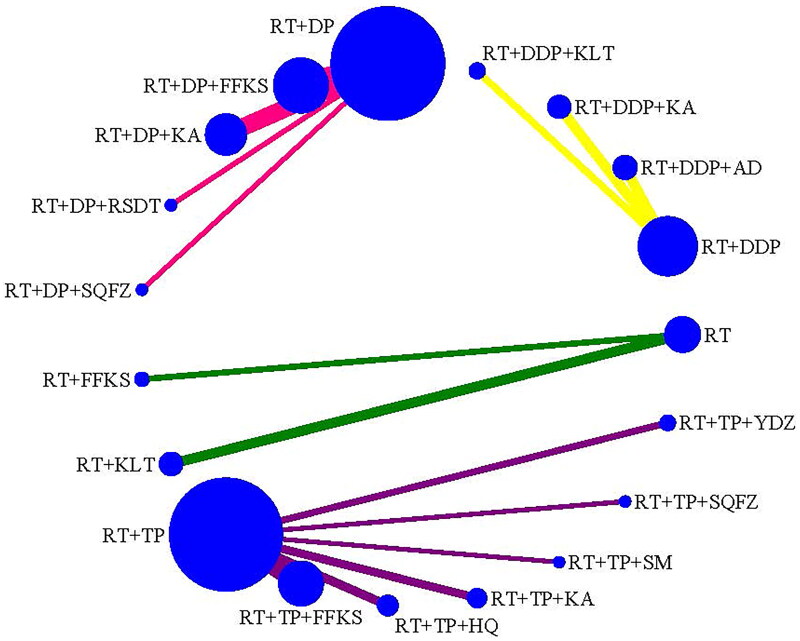
Network plot for the safety (GRR) of CMIs combined with RT or CCRT for cervical cancer.

GRR was reported in 25 studies (Yin et al. 2011; Zhou et al. 2011; Chen and Liu 2012; Chen and Zhang 2012; Wen et al. 2012; Chen 2013; Huang et al. 2014; Li et al. 2014; Zhong et al. 2014; Jiang et al. 2016; Mu 2016; Li 2017; Zhang 2017; Ma et al. 2018; Shao 2018; Wang et al. 2018; Cao 2019; Tian et al. 2019; Dong et al. 2020; Li et al. 2020; Liu et al. 2020; Song et al. 2020; Wang 2021; Ge et al. 2022; Peng and Li 2022) with 2171 cases, including 4 types of comparison (RT, RT + DDP, RT + DP, RT + TP) and 5 types of CMIs combination with RT, 4 types of CMIs combination with RT + DDP, 4 types of CMIs combination with RT + DP, and 6 types of CMIs combination with RT + TP (Figure 6).

The pooled results indicate that using RT + DDP + AD, RT + DDP + KA, and RT + DDP + KLT significantly reduced the GRR compared to RT + DDP. Similarly, RT + DP + FFKS and RT + DP + KA demonstrated a significant decrease in GRR compared to RT + DP (Table 3).

According to the SUCRA values for GRR, KLT was the most likely to be the best combination with RT. KLT was the most likely to be the best combination with RT + DDP. RSDT was the most likely to be the best combination with RT + DP, and KA was the most likely to be the best combination with RT + TP. The ranking of CMI interventions was as follows: RT + KLT (72.1%) > RT (51.7%) > RT + FFKS (26.2%); RT + DDP + KLT (90.9%) > RT + DDP + AD (62.1%) > RT + DDP + KA (46.6%) > RT + DDP (0.4%); RT + DP + KA (88.0%) > RT + DP + RSDT (64.9%) > RT + DP + KLT (46.9%) > RT + DP + FFKS (45.9%) > RT + DP (4.3%); and RT + TP + KA (58.6%) > RT + TP + SQFZ (57.7%) > RT + TP + SM (55.8%) > RT + TP + YDZ (52.5%) > RT + TP + FFKS (49.6%) >RT + TP + HQ (41.9%) > RT + TP (33.9%) (Table 4).

### Sensitivity analysis

A sensitivity analysis was performed to assess the impact of each study on the overall summary estimate, achieved by systematically omitting one study at a time. The findings indicate that excluding any single study did not significantly influence the outcomes (CER, KPS, LRR, GRR). This suggests that the results are statistically robust and reliable.

### Publication bias

**Figure 7.**
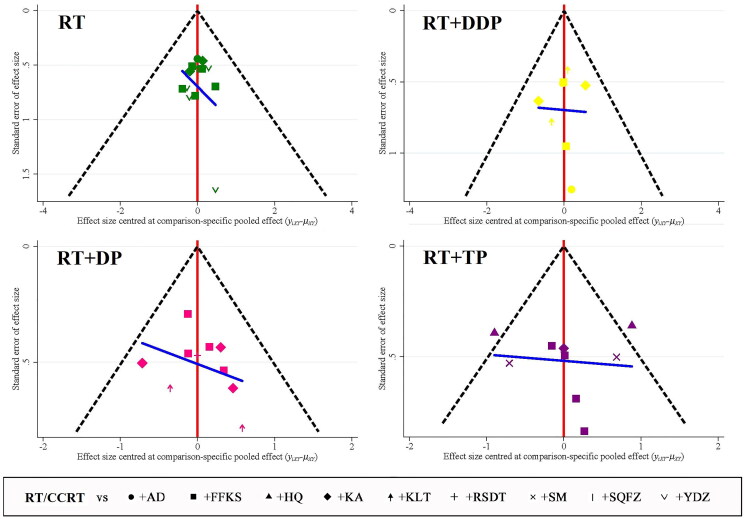
Publication bias (CER).

Comparison-adjusted funnel plots were generated for each outcome. Egger and Begg’s tests were conducted to assess the symmetry of the distributions. The results indicate some asymmetry in the CER funnel plots (Egger: *p*** **>** **0.05, Begg’s: *p*** **>** **0.05; Egger: *p*** **>** **0.05, Begg’s: *p*** **>** **0.05; Egger: *p*** **<** **0.05, Begg’s: *p*** **<** **0.05; Egger: *p*** **>** **0.005, Begg’s: *p*** **>** **0.05) as illustrated in Figure 7.

## Discussion

Cervical cancer poses a significant global public health challenge, with a particularly pronounced burden in many countries. In the management of cervical cancer, radiotherapy, especially brachytherapy, is the primary treatment for early-stage tumors with local risk factors. Additionally, cisplatin-based concurrent chemoradiotherapy is optimal for both efficacy and safety. TCM is crucial in China’s national work-related injury and maternity medical insurance, acting as a supplement or alternative to Western medicine. Chinese patent medicines play a vital supplementary role in cervical cancer treatment. A 2019 meta-analysis revealed that the combination of *Kangai* injection with cisplatin-based concurrent chemoradiotherapy exhibited higher efficacy (CER and KPS) and a lower incidence of side effects (LRR and GRR) compared to concurrent chemoradiotherapy alone (Cui et al. 2019).

Furthermore, a 2023 network meta-analysis demonstrated that *Fufangkushen* injection combined with paclitaxel plus cisplatin improved short-term efficacy and mitigated toxicity in cervical cancer treatment compared to paclitaxel plus cisplatin alone (Liu and Wang 2023). These published studies have strengthened the confidence of Chinese clinicians in using CMIs in conjunction with radiotherapy or cisplatin-based concurrent chemoradiotherapy for cervical cancer treatment. However, the optimal choice among CMIs combined with radiotherapy or cisplatin-based concurrent chemoradiotherapy for cervical cancer remains uncertain. Therefore, this network meta-analysis was undertaken to address this issue.

A total of 47 eligible studies comprising 4138 cases were included in this research, covering four types of comparison (RT, RT + DDP, RT + DP, RT + TP) and nine types of Chinese medicine injections (AD, FFKS, HQ, KA, KLT, RSDT, SQFZ, SM, YDZ). Based on the RR with a 95% CI and the SUCRA value, *Kanglaite* and *Kangai* are likely the optimal choices with radiotherapy for CER and KPS, respectively. Furthermore, *Kangai* and *Kanglaite* were likely optimal with radiotherapy and cisplatin for CER and GRR, respectively. *Kanglaite* was identified as the likely optimal choice with radiotherapy and docetaxel plus cisplatin for CER and *Kangai* for both KPS and GRR. Last, *Shenmai* and *Shenqifuzheng* were suggested as optimal choices with radiotherapy and paclitaxel plus cisplatin for CER and LRR, respectively. In summary, the optimal recommendation to combine Chinese medicine injections with radiotherapy or concurrent chemoradiotherapy depends on the specific conventional treatment used.

TCM, including Chinese medicine injections, exerts antitumor effects through two main aspects: health-strengthening (*Fu-Zheng*) and pathogen-eliminating (*Qu-Xie*). The key mechanism involves the regulation of the immune system in cancer patients (Wang et al. 2020; 2020). *Kanglaite* demonstrates inhibitory effects on various tumor cells, promoting tumor cell apoptosis by influencing related genes. For instance, it exhibits anti-triple-negative breast cancer effects by arresting the cell cycle and inhibiting cyclin-dependent kinase 1 (CDK1) dephosphorylation (Lu et al. 2008; Zhao et al. 2023). *Kangai*, on the other hand, exerts an antiproliferative function by inhibiting the interleukin (IL)-6/signal transducer and activator of transcription (STAT) 3 signaling pathway, subsequently inducing G1 phase arrest in gastric cancer cells (Zheng et al. 2022). *Shenmai* plays a role in remodeling the homeostasis of pro- and anti-angiogenic factors, promoting tumor vessel normalization, and enhancing drug delivery and antitumor effects. This insight into the pharmacological mechanisms of *Shenmai* in tumors is viewed from the perspective of vascular regulation (Cheng e al. 2021). When combined with chemotherapy, *Shenqifuzheng* exhibits better therapeutic efficacy than conventional chemotherapy alone, primarily through immunoregulation (Yang et al. 2017). Furthermore, animal research has indicated that *Shenqifuzheng* can alleviate fatigue symptoms in tumor-bearing mice by inhibiting pro-inflammatory cytokines produced by peripheral immune cells (Zhu et al. 2019).

However, when considering Chinese medicine injections as adjuvant treatment for cervical cancer, attention should be directed toward two critical aspects: multidrug resistance (MDR) chemotherapy and adverse drug reactions (ADRs) associated with CMIs. TCM is recognized for its crucial role in cancer treatment due to its low toxicity, high efficacy, safety, and potential to reverse MDR (Wei et al. 2022). In multidrug resistance, the existing literature suggests that *Kanglaite* can inhibit the expression of multidrug resistance-associated protein (MRP) 1 by suppressing the expression of PVT1 in gastric cancer (Zhang et al. 2017). Similarly, *Shenqifuzheng* has been reported to significantly enhance the efficacy of cisplatin in reducing tumor mass by regulating the expression of CD206 and CD86 (Yan et al. 2023). Regarding ADRs, an analysis of the spontaneous reporting system in Chinese Guangdong Province from 2003 to 2017 revealed that CMIs had a slightly lower percentage of serious ADRs and a notably higher percentage of unknown (new) ADRs compared to non-CMI injections. High-risk ADRs for CMIs included anaphylactic shock and anaphylactoid reactions (Li et al. 2019). Another analysis of Chinese Hubei province from 2014 to 2019 indicated that the age groups of children (0-10 years) and adult patients (41-80 years) had the highest rates of reporting ADRs for CMIs. Most ADRs occurred within one week, predominantly on the same day after receiving CMIs. Major concerns included anaphylactic shock, dyspnea, and anaphylactoid reactions, which were identified as the leading causes of death (Huang et al. 2021).

This research has several limitations. First, variations in characteristics such as age, disease stage, comparisons, interventions, and treatment courses may contribute to heterogeneity. Second, the absence of reported randomization methods in some literature and a lack of information on other biases could further contribute to increased heterogeneity. Furthermore, the exclusive focus on the literature from China may limit the generalizability and overall quality of the evidence. Last, the small sample size in some RCTs may lead to small study effects, affecting the robustness of the findings.

## Conclusions

Our network meta-analysis indicates that the use of Chinese medicine injections as an adjunctive treatment in combination with radiotherapy or concurrent chemoradiotherapy is associated with higher levels of efficacy and/or safety for cervical cancer compared to radiotherapy or concurrent chemoradiotherapy alone. The optimal recommendation for specific CMIs combined with radiotherapy or concurrent chemoradiotherapy varies depending on the conventional treatment scheme, with the guiding principle of maximizing patient benefits. However, it is crucial to acknowledge the poor quality of all included studies. To establish more robust conclusions, additional large-sample, multicenter, double-blind, and high-quality trials are needed to further confirm and update the existing evidence.

## Data Availability

All data generated or analyzed during this study are included in this published article, further inquiries can be made with the corresponding author(s).
